# A Massive Haemothorax Due to Pleural Extramedullary Haematopoiesis in a Patient With Myelodysplastic Syndrome/Myeloproliferative Neoplasm

**DOI:** 10.7759/cureus.99119

**Published:** 2025-12-13

**Authors:** Maha Theeb, Sarah Idris, Michael Sheaff, Kayleigh McCloskey

**Affiliations:** 1 Department of Haemato-Oncology, St Bartholomew’s Hospital, Barts Health NHS Trust, London, GBR; 2 Department of Histopathology, The Royal London Hospital, Barts Health NHS Trust, London, GBR

**Keywords:** extramedullary haematopoiesis, haemothorax, myelodysplastic/myeloproliferative neoplasm, myelodysplastic syndromes, pleural effusion, thoracic bleeding

## Abstract

Extramedullary haematopoiesis (EMH) refers to blood cell production outside the bone marrow, typically in reticuloendothelial organs such as spleen, liver, and lymph nodes, as a compensatory response to haematopoietic stress. We describe a rare case of recurrent haemothorax caused by pleural EMH in a female patient in her 60s with myelodysplastic/myeloproliferative neoplasm (MDS/MPN) not otherwise specified (NOS). She had recurrent presentations with transfusion-dependent anaemia, fevers, splenomegaly, and bone marrow morphology consistent with MDS/MPN. Over the course of her disease, she developed recurrent left-sided pleural effusions and haemothorax, initially managed with repeated drainage. Video-assisted thoracoscopic surgery (VATS) with pleural biopsy confirmed EMH with infiltration by myeloid precursors and megakaryocytes. Despite azacitidine therapy and supportive management, her condition progressed to multi-organ failure and death. The case highlights an unusual site of EMH and emphasises the need to consider EMH in the differential diagnosis of haemorrhagic pleural effusions in patients with malignant haematological disorders.

## Introduction

Extramedullary haematopoiesis (EMH) is the formation of blood cells outside of the bone marrow and is generally a compensatory response to inadequate marrow function or increased haematopoietic demand. EMH is classically associated with conditions such as haemolytic anaemia, thalassemia, and myeloproliferative disorders (including myelofibrosis and polycythaemia vera). While EMH predominantly occurs in the liver and spleen, it has been observed in unusual locations, such as the posterior mediastinum and paravertebral regions of patients with haemoglobinopathies, such as β-thalassaemia and hereditary spherocytosis [1]. Pleural EMH is markedly less common, though few published reports describe pleural effusions or haemothorax secondary to EMH in the context of chronic myeloid neoplasms. These include a case of primary pleural EMH in myelofibrosis presenting with massive haemothorax requiring surgical intervention [2], a case where pleural-fluid cytology alone identified trilineage haematopoietic elements consistent with EMH in a patient with underlying marrow disease [3], and a case of recurrent pleural effusions due to EMH that achieved complete resolution following low-dose palliative radiotherapy [4].

The diagnosis of EMH in patients with malignant haematological conditions is often made post-mortem, through the histological identification of megakaryocytes and other hematopoietic precursors [3,5]. When pleural effusion occurs in the context of EMH, it is believed to result from mechanical irritations or disruption between the pleural surface and adjacent pleural or paravertebral hematopoietic masses. Because these masses are highly vascular, their rupture can lead to haemothorax. Pleural EMH, as seen in this case, is exceedingly rare [2-5], and this report adds to the limited literature describing this unusual presentation.

## Case presentation

**Figure 1:**
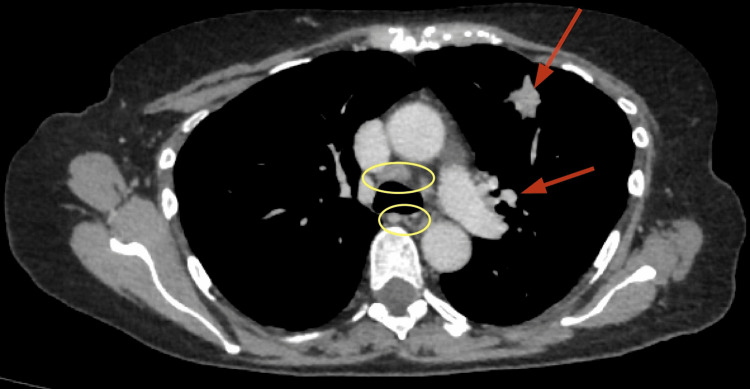
Contrast-enhanced CT chest showing multifocal consolidation and mediastinal lymphadenopathy on initial presentation Axial contrast-enhanced CT of the chest demonstrating multifocal pulmonary consolidation (red arrows) and enlarged mediastinal lymph nodes (yellow circles) on the mediastinal window. These findings were consistent with an inflammatory or infiltrative process, prompting exclusion of infection and further haematological workup.

A female in her 60s with a 20-pack-year smoking history presented with a two-month history of shortness of breath, fatigue, palpitations, and night sweats. Initial labs showed severe macrocytic anaemia (haemoglobin of 46 g/L) with a leucoerythroblastic blood film and elevated lactate dehydrogenase (LDH) with an otherwise normal full blood count. Haematinics and haemoglobin electrophoresis were normal, with mildly elevated reticulocytes and a positive direct antiglobulin test. Initial suspicion was for a haemolytic process, but blood results did not show biochemical evidence of ongoing haemolysis. A summary of laboratory findings is presented in Table 1. CT of the chest, abdomen, and pelvis (CT CAP) revealed multifocal consolidation and mediastinal lymphadenopathy (Figure 1).

**Table 1:** Laboratory results at presentation with reference ranges Abbreviations: MCV, mean corpuscular volume; WBC, white blood cell count; RBC, red blood cells;​​​​​​​ LDH, lactate dehydrogenase; DAT, direct antiglobulin test; INR, international normalised ratio; aPTT, activated partial thromboplastin time; U&Es, urea and electrolytes; LFTs, liver function tests; HbA, adult haemoglobin; HbA2, adult haemoglobin A2; HbF, fetal haemoglobin; HbS, sickle haemoglobin Note: Reference ranges are typical adult values and may vary by laboratory—verify against local ranges.

Parameter	Result (units)	Reference range
Haemoglobin	46 g/L (L)	115–165 g/L (F)
Haematocrit	0.16 (L)	0.36–0.46 (F)
MCV	103.8 fL (H)	80–100 fL
Reticulocytes	3.4% (H)	0.5–2.0%
WBC	8.4 × 10^9^/L	4.0–11.0 × 10^9^/L
Neutrophils	5.6 × 10^9^/L	2.0-7.0 × 10^9^/L
Monocytes	0.7 × 10^9^/L	0.2-1.0 × 10^9^/L
Immature granulocytes	1.8 × 10^9^/L (H)	≤0.10 × 10^9^/L
Nucleated RBC	1.5 × 10^9^/L (H)	0 × 10^9^/L
Platelets	318 × 10^9^/L	150–400 × 10^9^/L
LDH	594 U/L (H)	135–225 U/L
DAT	Positive (1+)	Negative
C-reactive protein (CRP)	33 mg/L (H)	<5 mg/L
Prothrombin time (PT)	10.5 s	9.5–13.5 s
Prothrombin ratio / INR	1.0	0.8–1.2
aPTT (ratio)	0.7 (L)	0.8–1.2
U&Es and LFTs	Within reference limits	—
Iron studies, folate, vitamin B12	Within reference limits	—
Haemoglobin electrophoresis (HbA, HbA2, HbF, HbS)	Normal pattern	—

**Figure 2:**
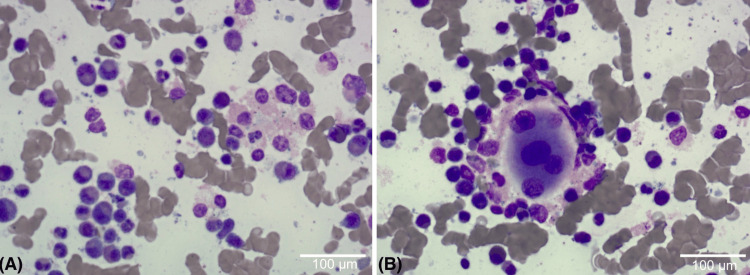
Bone marrow aspirate showing hypercellular marrow with dysplastic myeloid and megakaryocytic lineages (A) Hypercellular marrow with trilineage dysplasia and increased blasts. (B) Dysplastic myeloid precursors and abnormal megakaryocytes with nuclear irregularities. May–Grünwald–Giemsa stain, original magnification ×40; scale bar = 100 µm

She received blood transfusions and was referred for outpatient haematology follow-up. Over the following two months, she required repeated admission due to symptomatic anaemia and fever. During this period, she developed leucocytosis (white blood cell (WBC) count of 16.6 x 10^9^/L) with a monocyte count of 1.4 x 10^9^/L and progressive splenomegaly (135 mm → 160 mm). Bone marrow examination revealed a hypercellular marrow with tri-lineage dysplasia, a monocytoid expansion, and blast excess (9%). Cytogenetic analysis found trisomy 8, and a myeloid gene panel detected the following variants (with associated variant allele frequencies) - EZH2 (92%), BCOR (43%), STAG2 (40%), ASXL1 (45%), PTPN11 (32%), and TET 2 (46% and 40%). Bone marrow flow cytometry demonstrated approximately 7% myeloid progenitor cells (CD34⁺ CD117⁺ CD13⁺ CD33⁺ HLA-DR⁺ MPO⁺, cytoCD3⁻ CD19⁻ cytoCD79a⁻), consistent with a myeloid blast population. A proportion of neutrophils displayed aberrant CD56 expression. She was diagnosed with myelodysplastic syndrome/myeloproliferative neoplasm (MDS/MPN) not otherwise specified (NOS) in accordance with the WHO 2022 criteria [6]. Her monocyte count remained <10% of her total white cell count throughout, excluding CMML. Figure 2 shows the bone marrow aspirate.

On her third admission, she developed fever and left upper quadrant pain. A repeat CT CAP revealed small bilateral pleural effusions, new nodular changes, and progressive splenomegaly (21 cm). She tested positive for COVID-19 and required low-flow oxygen. Management included antibiotics (piperacillin/tazobactam), antivirals (nirmatrelvir/ritonavir), and dexamethasone. Her WBC count was elevated at 36.5 x 10^9^/L, prompting short-course cytoreductive therapy with hydroxycarbamide. Her case was reviewed at the haemato-oncology multidisciplinary team (MDT) meeting, where treatment with azacitidine was recommended (Eastern Cooperative Oncology Group (ECOG) performance status 3), with the possibility of a future stem cell transplant. Once her fevers subsided, she was commenced on azacitidine and was discharged after a six-week admission.

**Figure 3:**
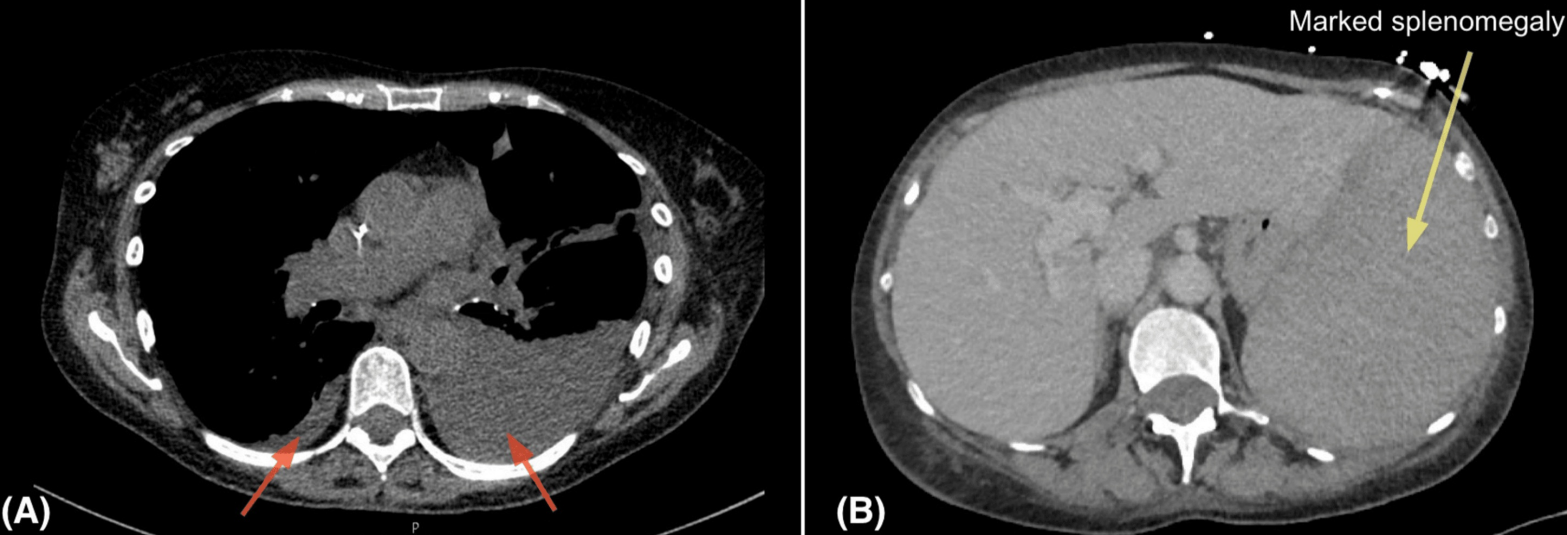
CT imaging demonstrating pleural effusions and splenomegaly (A) Axial high-resolution CT (HRCT) chest showing bilateral pleural effusions, larger on the left (red arrows). (B) Axial contrast-enhanced CT abdomen showing marked splenomegaly (yellow arrow).

Ten days later, she was readmitted with neutropenic fever and hypoxia. A high-resolution CT (HRCT) chest showed spiculated bilateral pleural effusions - larger on the left side - with centrilobular septal thickening (Figure 3A). A left pleural tap yielded blood-stained exudate with negative cytology and microbiology. An echocardiogram was performed to rule out a cardiac cause of effusion; this was normal. She was retreated for COVID-19 and improved, but the left-sided effusion reaccumulated within days, requiring a second drainage with similar findings. She improved and was discharged, but shortly, five days post-discharge, she was readmitted with fatigue, a fall, persistent fever, anaemia (Hb 43 g/L), deranged liver function tests (LFTs), and raised CRP (84 mg/L). Chest X-ray once again showed recurrent left pleural effusion. She received transfusions and intravenous antibiotics. Ultrasound and CT abdomen revealed hepatosplenomegaly with massive splenomegaly (24 cm) (Figure 3B).

**Figure 4:**
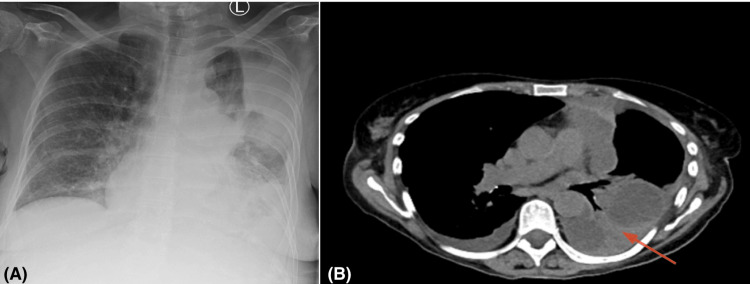
Chest radiograph and CT showing recurrent complex left pleural effusion despite prior video-assisted thoracoscopic surgery (VATS) drainage (A) Chest radiograph (PA) demonstrating near-complete opacification of the left hemithorax with a fluid meniscus and volume loss, compatible with a large recurrent pleural effusion. (B) Axial contrast-enhanced CT chest showing a complex, loculated left pleural effusion (red arrows) with compressed residual lung anteriorly.

The case was referred to the respiratory and thoracic team for VATS. Left VATS drainage and pleural biopsy were performed; fluid was blood-stained with negative microbiology. WBC rose sharply to 90 x 10^9^/L, and hydroxycarbamide was given. Antibiotics continued, and empirical antifungal therapy was started. Despite these interventions, her effusion recurred, and she rapidly deteriorated with progressive hypoxia (40-55% FiO_2_ on high-flow oxygen), tachypnoea, and high-grade fevers. A new chest radiograph and CT revealed a complex loculated pleural effusion (Figures 4A-4B).

**Figure 5:**
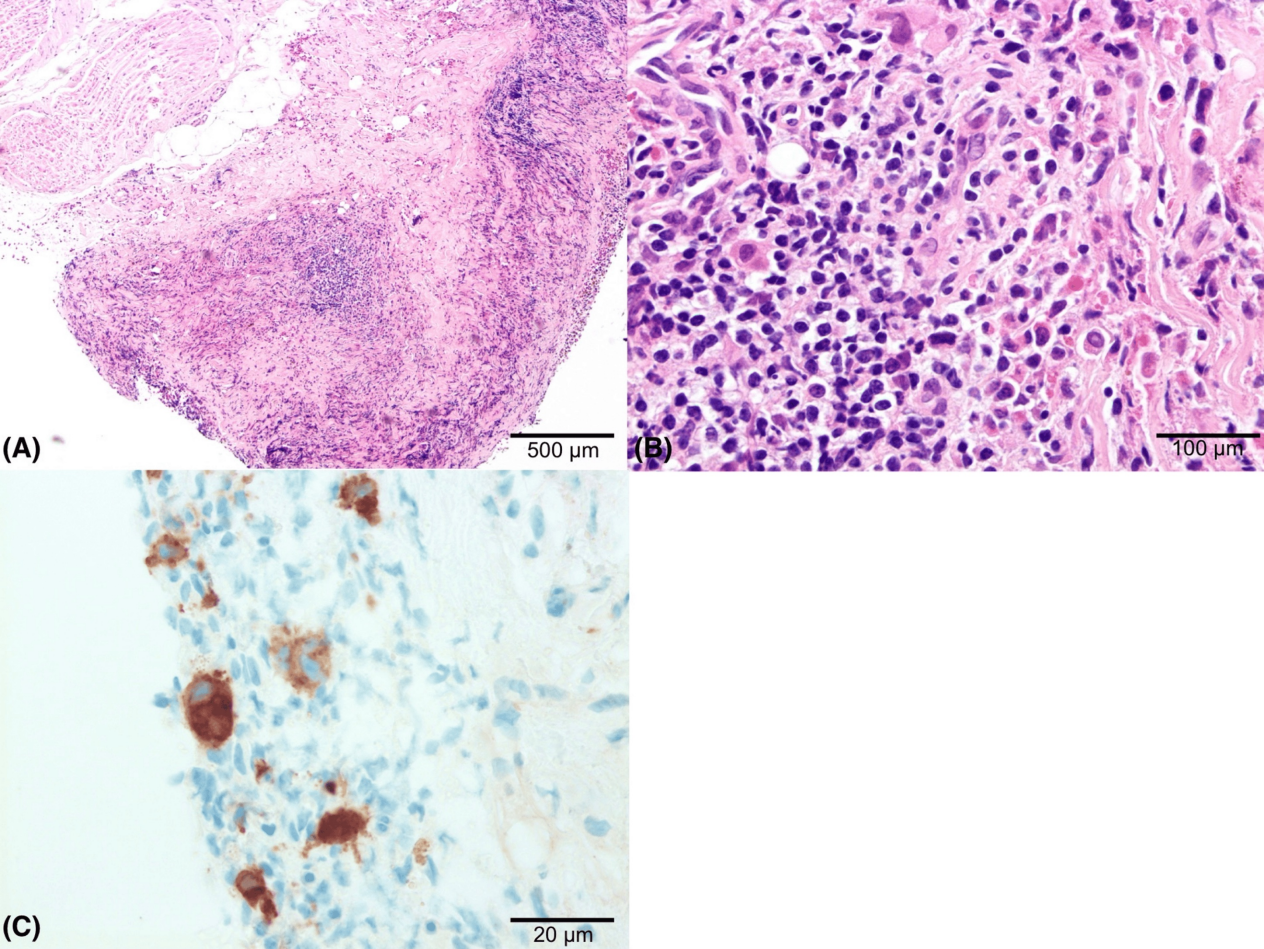
Pleural biopsy confirming extramedullary haematopoiesis (EMH) (A) Low-power H&E showing sheets of haematopoietic elements disrupting normal pleural architecture. (B) Higher-power H&E demonstrating infiltration by myeloid precursors with scattered megakaryocytes within pleural tissue. (C) Immunohistochemistry for a megakaryocytic marker highlighting megakaryocytes (brown). A: original magnification ×10; scale bar 500 µm. B: original magnification ×40; scale bar 100 µm. C: original magnification ×100; scale bar 20 µm

She underwent a second left VATS, converted to an anterolateral thoracotomy for extensive debridement. On the day of the procedure, pleural biopsy results from the previous VATS returned, confirming pleural extramedullary haematopoiesis with infiltration by myeloid precursors and megakaryocytes (Figures 5A-5C). Post-procedure, she became hemodynamically unstable with a significant drop in haemoglobin, unresponsive to transfusions. Chest X-ray showed a complete left-sided white-out due to massive haemothorax, necessitating re-exploration via thoracotomy the same day. Approximately one litre of clotted blood was evacuated from the pleural cavity, and multiple bleeding points on the lungs and pleura were identified but could not be surgically controlled. Despite intensive care, the patient’s condition deteriorated with multi-organ failure and ongoing bleeding (400 mL/hour). She died approximately 12 hours after the procedure, following a month-long final admission.

## Discussion

While EMH most commonly occurs in reticuloendothelial organs, such as the spleen and liver, intrathoracic involvement has been reported in the paravertebral space, posterior mediastinum, and lung parenchyma in patients with haemoglobinopathies, such as β-thalassaemia and hereditary spherocytosis [1]. Pleural surface involvement is an exceptionally rare phenomenon and is infrequently associated with significant pleural effusion or haemothorax, making this case of recurrent haemothorax secondary to pleural EMH particularly significant.

This case underscores the diagnostic complexities associated with EMH in patients with underlying MDS/MPN. The clinical presentation was characterized by recurrent pleural effusions, haemothorax, and respiratory deterioration without a clear infectious or malignant aetiology. Despite the presence of persistent fever, microbiological investigations remained negative throughout the disease course (with the exception of persistent COVID swab PCR positivity and *Klebsiella pneumoniae* in urine culture), and cytology revealed no malignancy, underscoring the limitations of standard investigations in such cases. Repeatedly negative microbiology, while not excluding infection, should prompt consideration of non-infectious differentials, such as EMH, especially in patients with haematological malignancies.

Definitive diagnosis in cases of pleural EMH often requires tissue sampling, as radiological findings are generally non-specific. Radiographic suspicion of this diagnosis can be bolstered by increased uptake of technetium-99m labelled tracers on bone marrow scintigraphy [2], and a few cases have documented the detection of EMH using FDG PET CT, typically identifying it as a benign mass with low SUV max values and normal tissue appearance [7]. In this case, a diagnosis was only established following VATS, which demonstrated infiltration of the pleura by myeloid precursors and megakaryocytes. This underscores the value of early tissue biopsy in persistent or unexplained pleural effusions when routine investigations are inconclusive.

Therapeutic options for EMH depend on the severity of clinical presentation. If the patient is asymptomatic and the anatomical location poses no risk, a "wait and watch" strategy may be adopted. In some cases, a conservative approach with supportive measures and blood transfusion may reduce haematopoietic drive. Cytoreductive therapies such as hydroxycarbamide and Janus kinase (JAK) inhibitors such as ruxolitinib have been used with variable success [5,8]. This approach is particularly beneficial for managing EMH secondary to haemoglobinopathies or congenital haemolytic anaemia [5]. Low-dose radiotherapy to the whole lung has proven to be an effective palliative treatment for pulmonary EMH [4].

The management of such complex presentations highlights the importance of a multidisciplinary approach. In this case, coordination between haematology, respiratory medicine, thoracic surgery, and critical care teams was crucial in guiding diagnostic and therapeutic decisions.

## Conclusions

Pleural extramedullary haematopoiesis is a rare but potentially life-threatening cause of recurrent pleural effusion and haemothorax in patients with haematological malignancies.

This case highlights the diagnostic challenges, the importance of early tissue biopsy when routine investigations are inconclusive, and the need for a coordinated multidisciplinary approach. Clinicians should consider pleural EMH in patients with unexplained haemorrhagic pleural effusions to enable timely recognition and intervention. Effective management requires a multidisciplinary approach, involving collaboration among haematologists, clinical oncologists, thoracic surgeons, respiratory specialists, and histopathologists, to facilitate more timely and targeted interventions.
